# Editorial: Sleep and circadian rhythms in plasticity and memory, volume II

**DOI:** 10.3389/fnsys.2024.1351714

**Published:** 2024-02-05

**Authors:** Jason R. Gerstner, H. Craig Heller, Sara J. Aton

**Affiliations:** ^1^Department of Translational Medicine and Physiology, Spokane, WA, United States; ^2^Steve Gleason Institute for Neuroscience, Washington State University, Spokane, WA, United States; ^3^Sleep and Performance Research Center, Elson S. Floyd College of Medicine, Washington State University, Spokane, WA, United States; ^4^Department of Biology, Stanford University, Stanford, CA, United States; ^5^Molecular, Cellular, and Developmental Biology, University of Michigan, Ann Arbor, MI, United States

**Keywords:** sleep, synaptic plasticity, cognition, circadian rhythm, learning, memory, phylogeny

Throughout the animal kingdom, cellular and molecular mechanisms that underlie sleep and circadian rhythms appear closely tied to cognitive processing and synaptic activity, suggesting a functional relationship that may be phylogenetically conserved. In this second volume of the Research Topic “*Sleep and circadian rhythms in plasticity and memory*” work highlights the importance of studies using both invertebrate and vertebrate models to tackle these complex interactions. Our understanding of these associations has important implications for overall human health and safety.

In their research article, Strobel et al. describe the role of sleep loss in cognitive performance of using a pairwise discrimination reversal learning model. Here, the authors report that when sleep deprived, rats struggle to adapt to reversed contingencies, while still completing a similar number of trials. These data suggest that the effects of sleep loss operate on cognitive rigidity, a behavior that describes resistance to adjustments of actions which previously led to a positive outcome, and no longer do.

In another original research article, Yin et al. further investigate the role of circadian disruption on memory formation using the fruit fly, *Drosophila melanogaster*. In this study, the authors characterize a post-training consolidation window using the olfactory avoidance-training paradigm (Tully and Quinn, 1985) when the circadian system is required for memory. Here, a post-training incubation of flies in dark:dark (DD) conditions, or induction of the bZIP transcription factor *vrille*, disrupts 3d long-term memory. Importantly, the authors ruled out the effects of these manipulations on learning and retrieval, suggesting that the role of the circadian system is necessary during this consolidation period following training. The authors posit that this 1–3 day post-training window likely operates alongside other temporal windows involving various transcriptional processes, including multiple core clock genes, and may differentiate their roles depending on the type of behavior (e.g., courtship suppression), which will require further investigation.

In the review article, “*In search for the retrievable memory trace in an insect brain*” Menzel discusses whether a “memory trace” exists beyond retrieval in the context of memory consolidation during sleep, and transfer of memory across brain areas. Here, Menzel goes into detail in anatomical comparison of brain circuits involved in olfactory processing between *Drosophila* and the honeybee. Here Menzel describes the antennal lobe to mushroom body circuitry where synaptic connections form essential “functional units of boutons” that are convergence sites of conditional stimulus and unconditional stimulus pathways. These sites therefore represent adaptable physical and anatomical units of a memory trace at the level of the synapses and suggests future studies using these invertebrate models to capture observational patterns of encoded memories.

In another review article, “*The role of calcium and CaMKII in sleep*” Wang et al. provide an overview of work on calcium signaling and Ca2+/calmodulin-dependent protein kinase II (CaMKII) in sleep regulation. Here, the authors describe the Ca2+-dependent hyperpolarization pathway in sleep as a signaling molecule, leading to CaMKII α and β as sleep-promoting kinases. The authors also discuss how these mechanisms might also be differentiated between cell types (i.e., neurons vs. glia) and how there may be phylogenetic conservation of certain subcellular processes between species (e.g., rodent models and invertebrates, such as *Drosophila*). Wang et al. suggest that these and other kinases associated with sleep regulation may greatly influence the global phosphorylation status within brain cells and likely connect multiple signaling pathways to sleep-related physiological function.

In their perspective piece, Gerstner et al. challenge traditional models of circadian and sleep regulatory processes that create distinct molecular and cellular mechanisms to distinguish these behaviors, and instead provide an alternative view which integrates them in a functional unit that combines the blood brain barrier (BBB), astrocytes, and neurons. Here, the authors propose that the transcript of the core circadian clock driven output gene, the brain fatty-acid binding protein (Fabp7), is trafficked to the fine-peri-synaptic astrocytic processes where it is locally processed by activity-dependent local translation to participate in wake-promoting lipid exchange between cells. Therefore, experience-dependent plasticity-related processes associated with wakefulness drive lipid-signaling cascades from neurons to glia, where glia can scavenge and store toxic lipid waste products in lipid droplets, which can be used for β-oxidation energy utilization when glycogen stores are depleted, for example, during sleep. Fabp7 may in turn influence BBB permeability, which varies based on time-of-day and sleep/wake state, to regulate the uptake of important nutrients, and release of toxic molecules and waste products. Together, this perspective places Fabp7 in astrocytes as an important functional node to integrate sleep/wake status with the circadian system, together with the bioenergetics and metabolic demands of plasticity-related processes important for cognitive function.

This Research Topic highlights the importance of utilizing multiple models and species to increase our knowledge of the relationship between sleep, clocks, and cognitive function. These and future studies extending from this and previous work (Gerstner et al., 2015) will help to provide valuable inroads toward our understanding of phylogenetically conserved mechanisms integrating these complex behaviors.

## Author contributions

JG: Writing – original draft. HH: Writing – review & editing. SA: Writing – review & editing.
